# The association between stress hyperglycemia ratio and 1-year outcomes in patients with acute myocardial infarction: a retrospective large sample cohort study

**DOI:** 10.3389/fendo.2025.1586541

**Published:** 2025-04-15

**Authors:** Ning Yan, Peng Wu, Zhengjun Zhang, Mohan Wang, Juan Ma, Ali Ma, Dapeng Chen, Xueping Ma, Xiaocheng Li

**Affiliations:** ^1^ Heart Centre and Department of Cardiovascular Diseases, General Hospital of Ningxia Medical University, Yinchuan, China; ^2^ First Clinical College, Ningxia Medical University, Yinchuan, China; ^3^ Institute of Basic Medical Sciences, Xi’an Medical University, Xi’an, China

**Keywords:** acute myocardial infarction, major adverse cardiovascular and cerebrovascular events (MACCE), stress hyperglycemia, stress hyperglycemia ratio (SHR), long-term prognosis

## Abstract

**Background:**

The Stress Hyperglycemia Ratio (SHR) is associated with poor outcomes in coronary artery disease patients, but its link to Acute Myocardial Infarction (AMI) prognosis is unclear. This study explores the relationship between SHR and 1-year outcomes after AMI using a large cohort analysis.

**Methods:**

This retrospective study enrolled 4012 AMI patients from General Hospital of Ningxia Medical University(2016-2019). These patients were stratified into three distinct groups according to the tertiles of the SHR: Group T1 (SHR < 0.90, n=1337), Group T2 (0.90 ≤ SHR < 1.11, n=1337), and Group T3 (SHR ≥ 1.11, n=1338). All patients were clinically followed for 1-years to collect major adverse cardiovascular and cerebrovascular events (MACCE). After controlling for different confounding factors, cox regression models and restricted quadratic splines were used to investigate the relationship between SHR and 1-years clinical outcomes.

**Results:**

During the 1-year follow-up, 229 all-cause deaths were recorded, yielding a mortality rate of 5.71% (n=229). Additionally, 861 MACCE were recorded, yielding a MACCE rate of 21.46%. After adjusting for covariates, SHR was found to be significantly associated with 1-year MACCE [hazard ratio (HR) = 2.18; 95% confidence interval (CI) = 1.64-2.89; *P* < 0.001] and all-cause mortality (HR = 3.11; 95% CI = 1.77-5.46; *P* < 0.001) in patients with AMI, and the T3 group exhibited a higher risk of 1-year MACCE (HR = 1.67; 95% CI = 1.34-2.09; *P* < 0.001) and all-cause mortality (HR = 1.67; 95% CI = 1.02-2.73; *P* =0.042) compared with T1 group. A J-shaped relationship was observed between SHR and 1-year MACCE as well as all-cause mortality, showing a turning point at 0.87. Beyond this threshold, the hazard ratio for 1-year MACCE was 2.64 (95% CI: 1.91-3.65), and for all-cause mortality was 4.26 (95%: CI 2.30-7.86). The results remained consistent across subgroup.

**Conclusion:**

SHR is significantly and positively associated with one-year clinical outcomes in patients with AMI. Furthermore, there is a specific non-linear association between SHR and MACCE and all-cause mortality (both inflection point 0.87). Interventions aimed at reducing SHR levels below 0.87 through medication management have the potential to significantly improve outcomes.

## 1 Introduction

The utilization of reperfusion strategies and the enhancement of regional coordinated treatment systems have led to a notable decrease in acute-phase mortality in patients diagnosed with acute coronary syndrome (ACS) (1, 2). However, despite these advancements, AMI patients who receive emergency percutaneous coronary intervention (PCI) still remain susceptible to short-term major adverse cardiac and cerebrovascular events (MACCE), which can significantly affect their quality of life (3). Early identification of high-risk patients and the management of relevant risk factors are beneficial for improving the prognosis of AMI patients (4).

Stress-induced hyperglycemia (SIH) arises from a neurohormonal cascade triggered by acute physiological stress, characterized by increased catecholamines, cortisol, and cytokines. These mediators promote hepatic gluconeogenesis, impair insulin secretion, and induce peripheral insulin resistance, leading to transient hyperglycemia independent of chronic glycemic status (5, 6). Nonetheless, Furthermore, the literature presents a divergent view on the prognostic value of stress-induced hyperglycemia in patients with AMI, with findings varying across different studies (7, 8). One plausible interpretation could be that the elevated blood glucose levels observed in patients upon admission may be interpreted as stress-induced hyperglycemia. The blood glucose levels observed in hospitalized individuals can be impacted by both the stress response and the initial glucose levels, potentially complicating the accurate assessment of SIH (9). To address this issue, Roberts et al. in 2015 proposed Stress Hyperglycemia Ratio (SHR) as an innovative metric (10), which adjusts stress glucose levels by baseline glucose levels, providing a more accurate depiction of glucose level fluctuations under stress conditions.

Recently, the stress hyperglycemia ratio (SHR) has been suggested as a superior measure of relative stress hyperglycemia, which is calculated from admission glucose adjusted for chronic glycemic status using glycosylated hemoglobin (HbA1c) (11, 12).

Recent evidence from Ben Hu et al. demonstrates a J-curve relationship between stress hyperglycemia ratio and short-term mortality in AMI patients, revealing an inflection point at SHR=0.9 that predicts increased 90-day (HR=1.89, 95%CI:1.32-2.71) and 180-day (HR=2.04, 95%CI:1.45-2.87) mortality risks (13). An analysis of 904 cardiogenic shock patients from the MIMIC-IV database identified a significant positive correlation between elevated SHR and mortality in cardiogenic shock(CS) patients. Higher SHR tertiles were associated with increased 30-day (HR=2.140, 95%CI:1.522-3.008) and 360-day (HR=1.495, 95%CI:1.157-1.931) risks, particularly amplified in acute myocardial infarction subgroups (interaction *p*<0.01) *(* 14). Although those studies have established the significantly association between SHR and an increased risk of all-cause mortality in acute myocardial infarction, but two critical knowledge gaps persist. First, emerging evidence suggests potential non-linear biological effects of glycemic dysregulation, yet no large-scale study has systematically examined the associations between SHR and long-term outcomes. Second, the clinically actionable threshold for SHR optimization remains undefined, as highlighted in a recent meta-analysis (15). Nevertheless, studies on the association between SHR and the occurrence of MACCE within one year in post-AMI patients are limited. The primary objective of this study is to explore the link between SHR and 1-year MACCE and all-cause mortality in individuals with AMI, with a specific emphasis on non-linear associations and threshold effects, utilizing a large-scale cohort investigation.

## 2 Methods

### 2.1 Study design and patients

This study was conducted in accordance with the Declaration of Helsinki and was approved by the Ethics Review Committee of General Hospital of Ningxia Medical University (Approval No. No:2020-771). This study has been registered and published by the Chinese Clinical Trials Registry (registration number: ChiCTR2100043359). As this was a retrospective cohort study and the follow-up was performed by phone, the ethics committee permitted verbal consent.

**Figure 1:**
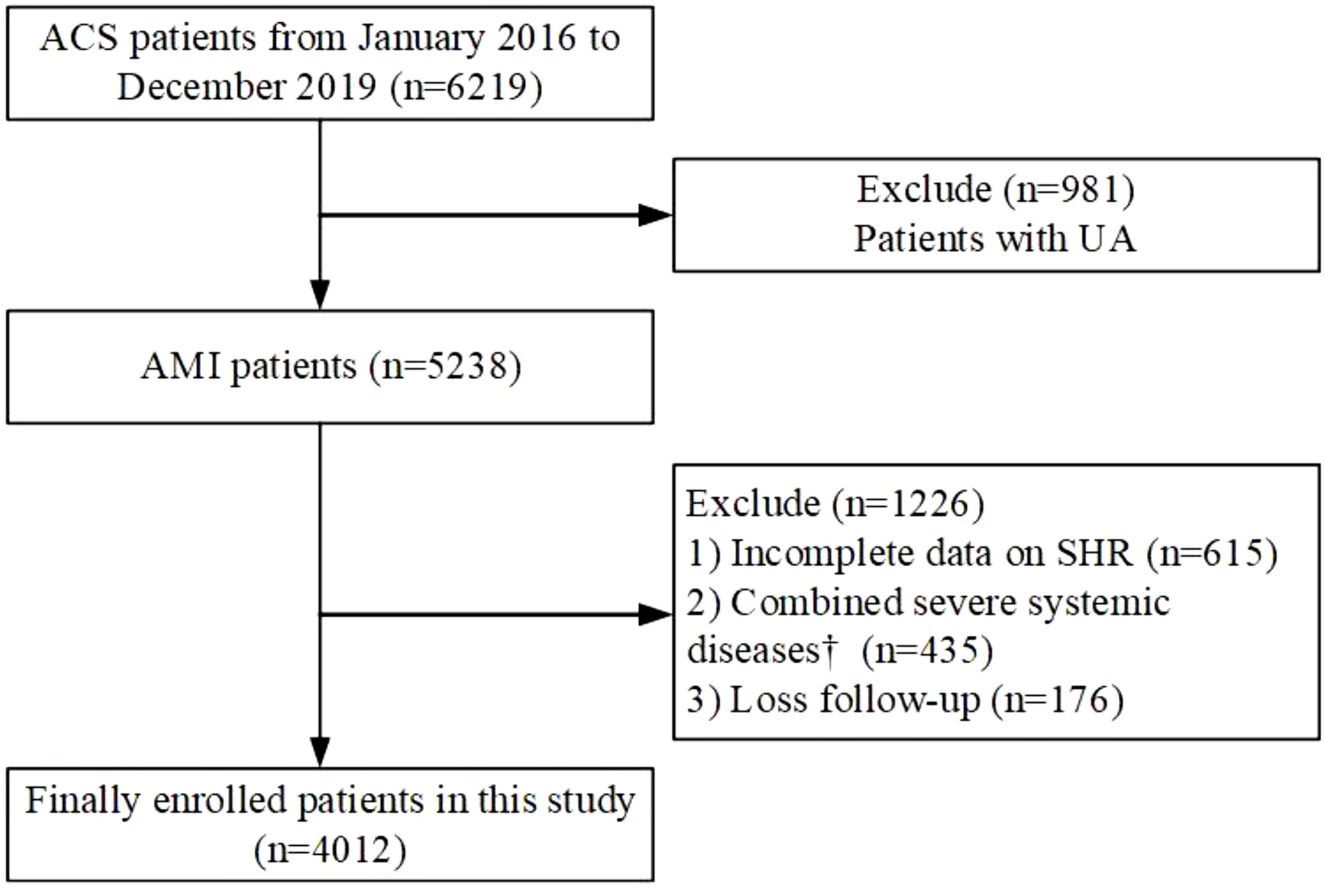
Flowchart illustrating the selection process of study participants. ACS, acute coronary syndrome; AMI, acute myocardial infarction; UA, unstable angina; SHR, Stress hyperglycemia ratio. † Including severe valvular heart disease, decompensated heart failure, non-ischemic dilated cardiomyopathy, severe renal or hepatic disease, acute infection and/or inflammation, malignancy, hematologic disease, autoimmune disease.

This study was a single-center, retrospective large cohort study. From January 1, 2016 to December 30, 2019, 6219 consecutive patients with the manifestation of ACS underwent coronary angiography at General Hospital of Ningxia Medical University were enrolled in this study. Exclude criteria:1) Diagnose with UA(n=981); 2) Incomplete data on SHR (n=615); 3) Combined severe systemic diseases(n=435); 4) Loss follow-up(n=176). Patients were followed up from March 2023 to September 2023 by telephone (**Figure 1**). Finally, 4012 patients were included in the final analysis. Patients were divided into three groups according to SHR tertile.

### 2.2 Data measurement and definitions

Three trained data abstractors collected information from medical records using standardized protocols. After carefully examination of patients’ electronic health records, we are starting to gather essential data included demographic information[age, gender, body mass index (BMI), admission diagnosis, GRACE score (GS)], cardiovascular risk [DM, hypertension, prior cerebrovascular disease (CVD), hyperlipemia, and prior coronary artery disease (CAD), current smoking habits], data related to angiography and basic cardiovascular medication information [antiplatelet drugs, statins, beta-blockers, angiotensin-converting enzyme inhibitors (ACEI)/angiotensin receptor blockers (ARB)]. Systolic blood pressure (SBP), diastolic blood pressure (DBP), and heart rate (HR) on hospital admission were recorded. The left ventricular ejection fraction (LVEF) was measured using the two-dimensional modified Simpson’s method, providing essential data for evaluating cardiac function. The calculation for BMI involves dividing weight by height squared, with both measurements in metric units. The admission blood glucose (ABG) is the initial random serum glucose measured within the first 24 hours of admission. Fasting blood samples were collected from the cubital vein for measurement of hemoglobin A1c (HbA1c), high-sensitivity C-reactive protein (hs-CRP), peak cardiac troponin I (cTnI), serum creatinine (Scr), uric acid (UA), total cholesterol (TC), triglycerides (TG), high-density lipoprotein cholesterol (HDL-C), low-density lipoprotein cholesterol (LDL-C), and D-dimer (D-D). cTNI levels was measured using chemiluminescent immunoassay (VITROS5600, Johnson & Johnson, USA), and HbA1c, hs-CRP, Scr, UA, TC, TG, HDL-C, and LDL-C, D-D levels were analyzed using ADVIA^®^ Chemistry XPT system (SIEMENS, Germany) at the central laboratory of General Hospital of Ningxia Medical University.

SHR was calculated using the following equation: SHR= ABG (mmol/L)/[1.59 × HbA1c (%)-2.59] (10).

Diagnosis of AMI (including STEMI and NSTEMI) was based on the 2023 ESC Guidelines, incorporating symptoms, electrocardiographic changes, and cardiac biomarker elevation (16). The identification of diabetes mellitus (17) was based on either the self-reported use of antidiabetic medications or elevated blood glucose readings, characterized by casual blood glucose levels of 11.1mmol/L or higher, fasting blood glucose levels of 7.0mmol/L or higher, or 2-hour postprandial levels exceeding 11.1mmol/L following a 75 g oral glucose tolerance test. Hypertension was identified through a consistent record of blood pressure readings of 140/90mmHg or above, or the ongoing use of antihypertensive medication (18). According to coronary angiography results, the severity of CAD was evaluated by the Gensini Score (19), and multivessel disease was defined as ≥ 50% diameter stenosis in at least 2 major coronary arteries. Smoking was defined as individuals who had engaged in smoking within the past ten years. Hyperlipidemia was defined as LDL-C concentrations ≥ 3.4 mmol/L, HDL-C < 1.0 mmol/L, TC≥ 1.7 mmol/L, or patients who were taking lipid-lowering medication (20).

### 2.3 Follow-up and endpoints

Following discharge, patients were required to attend follow-up evaluations at 1, 3, 6, and 12 months, with additional annual appointments scheduled either via phone calls or in-person visits to the clinic. During the follow-up duration, trained professionals meticulously recorded any clinical events that occurred. The primary endpoint was 1-year MACCE, which encompassed all-cause mortality, nonfatal myocardial infarction (MI), rehospitalization for angina, rehospitalization for heart failure, and nonfatal stroke. The study endpoint was evaluated based on the time taken for the occurrence of the first event. All-cause mortality referred to death regardless of the cause. Nonfatal myocardial infarction defined as myocardial necrosis without resulting in death. It is accompanied by symptoms of ischemia, abnormal levels of myocardial markers, ST segment changes, or Q wave changes. Rehospitalization for angina and heart failure define as admission for treatment due to a recurrence of angina or heart failure. Nonfatal stroke was defined as disabling vascular brain injury caused by cerebral ischemia or hemorrhage. Clinical events were adjudicated by an independent endpoint committee using standardized diagnostic criteria, including electrocardiographic changes, biomarker elevations (e.g., troponin I), and imaging evidence. Hospitalization records were cross-validated with ICD-10 codes (I21 for MI, I50 for heart failure, I63 for stroke).

Medication adherence: Post-discharge medication use (including antiplatelet agents, statins, beta-blockers, ACEI/ARB, and glucose-lowering therapies) was recorded during follow-up visits. Adherence was defined as ≥80% compliance with prescribed regimens, verified via pharmacy records and patient self-report.

### 2.4 Statistical analysis

In this study, all statistical analyses were performed with R (the R Foundation, Vienna, Austria) and Empower (X & Y Solutions, Boston, MA, USA). All tests were 2-sided, and a *P* < 0.05 was considered statistically significant.

For continuous variables, mean ± standard deviation (SD) was used for statistical description if they met normal distribution; independent samples t-test was used for inter-group comparison. the median (25-75%) was used for description if the variables did not meet normal distribution; and the rank sum test was used for inter-group comparison. For counting data, the number of cases (%) was used to describe it, the chi-square test was used for comparison between groups, and Fisher’s exact probability was used when the chi-square test was not satisfied. Continuous data were compared using the Kruskal-Wallis’s test or one-way analysis of variance, and categorical data were compared using the chi-squared test. A multivariable Cox proportional hazards regression models were used to calculate hazard ratios (HRs) between SHR and MACCE. We used three levels of adjustment: Model 1 was adjusted for age, sex, BMI; Model 2 was adjusted for age, sex, BMI, DM, hypertension, previous CVD, hyperlipemia, prior CAD, current smoking; and Model 3 was adjusted age, sex, BMI, DM, hypertension, previous CVD, hyperlipemia, prior CAD, current smoking, hs-CRP, cTNI, Scr, PPCI, ACEI/ARB, Gensini Score, UA, D-D.

To explore the potential non-linear association between SHR and mortality, Cox proportional hazards regression models with restricted cubic spline (RCS) was employed, with knots placed at the 5th, 35th, 65th, and 95th percentiles of SHR. In the RCS model, we also adjusted for confounding factors: age, sex, BMI, DM, hypertension, previous CVD, hyperlipemia, prior CAD, current smoking, hs-CRP, cTNI, Scr, PPCI, ACEI/ARB, GS, UA, D-D. If the relationship was nonlinear, we estimated the threshold value and selected the inflection point with the highest likelihood. We used a two-piecewise Cox proportional hazards model on both sides of the inflection point to investigate the association between SHR and the MACCE or all-cause mortality risk.

Subgroup analyses of different subgroups (age, sex, DM, hypertension, smoking status, LVEF statues, current smoking statues, and lesion vessel number) were performed using stratified Cox proportional hazards regression models. In addition to stratification factors, we adjusted for age, sex, BMI, DM, hypertension, previous CVD, hyperlipemia, prior CAD, current smoking, hs-CRP, cTNI, Scr, PPCI, ACEI/ARB, Gensini Score, UA, D-D except the subgroup variable. To assess the presence of an interaction term, we used likelihood ratio tests in models with and without an interaction term.

## 3 Results

### 3.1 Participants’ characteristics

A total of 4012 patients with AMI were included in the final analysis. The mean age of the study population was 61.32 ± 11.75 years, and 3025 (75.4%) patients were male. Patients were divided into three groups according to the SHR tertiles. Patient sex, HR, SBP, STEMI, GRACE score, DM, prior CVD, Current smoking, ABG, HbA1c, hs-CRP, cTNI, SCR, TG, LDL-C, TC, LVEF, use of ACEI/ARB, PPCI, Gensini score, minimal lumen area, number of stents were significantly different between the three groups (all *P* < 0.05) (**Table 1**). Patients in the T3 group were more likely to have higher ABG and HBA1C, and a history of DM, prior CVD and a diagnosis of STEMI (**Table 1**).

**Table 1:** Baseline characteristics of study population according to the tertiles of SHR (N =4012).

Variable	Total (n=4012)	Tertile 1 (n=1337)	Tertile 2 (n=1337)	Tertile 3 (n=1338)	*P-value*
General conditions					
Age(years)	61.32 ± 11.75	61.08 ± 11.73	61.02 ± 11.89	61.85 ± 11.62	0.094
Male, n (%)	3025 (75.40%)	1029 (76.96%)	1030 (77.04%)	966 (72.20%)	0.004
BMI(Kg/m2)	24.39 ± 4.47	24.33 ± 4.84	24.25 ± 4.34	24.60 ± 4.19	0.072
HR (bpm)	81.03 ± 16.15	78.61 ± 14.95	80.62 ± 15.70	83.86 ± 17.29	<0.001
SBP (mmHg)	121.96 ± 22.15	122.74 ± 21.35	122.64 ± 21.89	120.50 ± 23.10	0.008
DBP (mmHg)	75.97 ± 14.12	76.05 ± 13.59	76.46 ± 14.01	75.39 ± 14.71	0.152
STEMI, n (%)	3053 (76.12%)	959 (71.73%)	1044 (78.14%)	1050 (78.48%)	<0.001
GRACE	153.73 ± 35.68	149.10 ± 32.28	151.11 ± 32.77	160.97 ± 40.28	<0.001
Risk factor, n (%)					
DM	1196 (29.82%)	369 (27.60%)	302 (22.60%)	525 (39.24%)	<0.001
Hypertension	2160 (53.84%)	717 (53.63%)	710 (53.10%)	733 (54.78%)	0.672
Prior CVD	508 (12.66%)	164 (12.27%)	147 (10.99%)	197 (14.72%)	0.013
Hyperlipemia	1642 (40.93%)	555 (41.51%)	539 (40.31%)	548 (40.96%)	0.82
Prior CAD	579 (14.43%)	209 (15.63%)	174 (13.01%)	196 (14.65%)	0.151
Current smoking	2399 (59.80%)	850 (63.58%)	803 (60.06%)	746 (55.75%)	<0.001
Current drinking	796 (19.84%)	265 (19.82%)	277 (20.72%)	254 (18.98%)	0.531
Laboratory test					
ABG (mmol/L)	8.18 ± 3.77	5.99 ± 1.84	7.36 ± 2.32	11.18 ± 4.41	<0.001
HBA1C (%)	6.51 ± 1.55	6.58 ± 1.55	6.26 ± 1.40	6.69 ± 1.66	<0.001
hs-CRP (mg/L)	25.82 ± 38.27	25.63 ± 38.14	23.97 ± 37.09	27.75 ± 39.43	0.007
cTNI (ng/L)	18.32 ± 20.64	14.92 ± 18.75	19.29 ± 20.91	20.72 ± 21.69	<0.001
SCR (mmol/L)	73.75 ± 23.05	73.98 ± 22.32	72.10 ± 20.92	75.18 ± 25.57	0.023
UA (mmol/L)	341.85 ± 101.57	341.26 ± 100.75	340.62 ± 99.04	343.69 ± 104.89	0.824
TG (mmol/L)	1.72 ± 1.30	1.70 ± 1.20	1.65 ± 1.36	1.81 ± 1.33	<0.001
TC (mmol/L)	4.18 ± 1.03	4.07 ± 1.03	4.24 ± 1.01	4.22 ± 1.02	<0.001
LDL (mmol/L)	2.17 ± 0.69	2.13 ± 0.68	2.23 ± 0.71	2.16 ± 0.69	0.002
HDL (mmol/L)	0.93 ± 0.23	0.92 ± 0.23	0.93 ± 0.22	0.94 ± 0.24	0.303
D-D (*μ*g/mL)	0.66 ± 0.96	0.74 ± 1.65	0.61 ± 0.96	1.04 ± 3.27	<0.001
LVEF (%)	51.83 ± 10.29	52.90 ± 10.48	51.98 ± 9.89	50.63 ± 10.37	<0.001
SHR	0.31-2.19	0.31-0.90	0.90-1.11	1.11-2.19	<0.001
Medications, n (%)					
Antiplatelet drugs	1.00 ± 0.00	1.00 ± 0.00	1.00 ± 0.00	1.00 ± 0.00	0.368
Statins	3995 (99.58%)	1330 (99.48%)	1333 (99.70%)	1332 (99.55%)	0.661
Beta-blockers	2967 (73.95%)	1005 (75.17%)	993 (74.27%)	969 (72.42%)	0.256
ACEI/ARB	1678 (41.82%)	608 (45.47%)	563 (42.11%)	507 (37.89%)	<0.001
Angiographic characteristics					
Primary PCI, n (%)	2395 (59.70%)	691 (51.68%)	869 (65.00%)	835 (62.41%)	<0.001
Gensini score	76.69 ± 45.23	73.46 ± 44.97	77.21 ± 45.77	79.43 ± 44.76	<0.001
Left-domain, n (%)	238 (6.40%)	70 (5.63%)	82 (6.46%)	86 (7.12%)	0.32
Infarction related artery					
LM	36 (1.24%)	15 (1.53%)	6 (0.60%)	15 (1.64%)	0.075
LAD	1679 (51.44%)	534 (48.33%)	567 (50.94%)	578 (55.26%)	0.005
LCX	524 (17.46%)	173 (17.08%)	204 (19.62%)	147 (15.51%)	0.051
RCA	1194 (37.80%)	397 (37.59%)	407 (37.44%)	390 (38.39%)	0.893
Minimal lumen area (mm²)	2.12 ± 0.85	2.25 ± 0.78	2.10 ± 0.82	2.01 ± 0.91	0.003
Total stent length (mm)	43.40 ± 27.63	44.78 ± 29.27	43.47 ± 27.58	43.06 ± 25.86	0.174
Calcification severity, n (%)					0.032
Mild	1562 (38.93%)	560 (41.88%)	525 (39.27%)	477 (35.65%)	
Moderate	892 (22.23%)	280 (20.94%)	298 (22.29%)	314 (23.47%)	
Severe	327 (8.15%)	98 (7.33%)	112 (8.38%)	117 (8.74%)	
Diffuse lesions, n (%)	1154 (28.76%)	378 (28.27%)	370 (27.67%)	406 (30.34%)	0.278
Chronic total occlusion, n (%)	327 (8.15%)	98 (7.33%)	112 (8.38%)	117 (8.74%)	0.441
NDV					0.278
MVD, n (%)	2858 (71.24%)	959 (71.73%)	967 (72.33%)	932 (69.66%)	
SVD, n(%)	1154(28.76%)	378(28.27%)	370(27.67%)	406(30.34%)	
Stent length, mm	44.40 ± 27.63	46.78 ± 29.27	43.47 ± 27.58	43.06 ± 25.86	0.014
Number of stents, n (%)	1.72 ± 0.96	1.78 ± 0.99	1.69 ± 0.97	1.68 ± 0.92	0.033

Values are presented as mean ± SD, or number (%), or median (interquartile range).

SHR, stress hyperglycemia index; TG, triglyceride; HDL-C, high density lipoprotein cholesterol; BMI, body mass index; SBP, systolic blood pressure;

DBP, diastolic blood pressure; CAD, coronary artery disease; STEMI, ST-segment elevation myocardial infarction; ABG, fasting plasma glucose; TC, total cholesterol;

LDL-C, low-density lipoprotein cholesterol; D-D: D-dimer; LVEF, left ventricular ejection fraction; LM, left main coronary artery; LAD, left anterior descending; LCX, left circumflex coronary artery; RCA, right coronary artery; ACEI/ARB, angiotensin-converting enzyme inhibitor/angiotensin receptor blocker. NDV, number of diseased vessels; SVD, single vessel disease; MVD, multivessel disease; IRA, Infarction related artery.

### 3.2 Association between SHR and 1-year clinical outcomes

**Figure 2:**
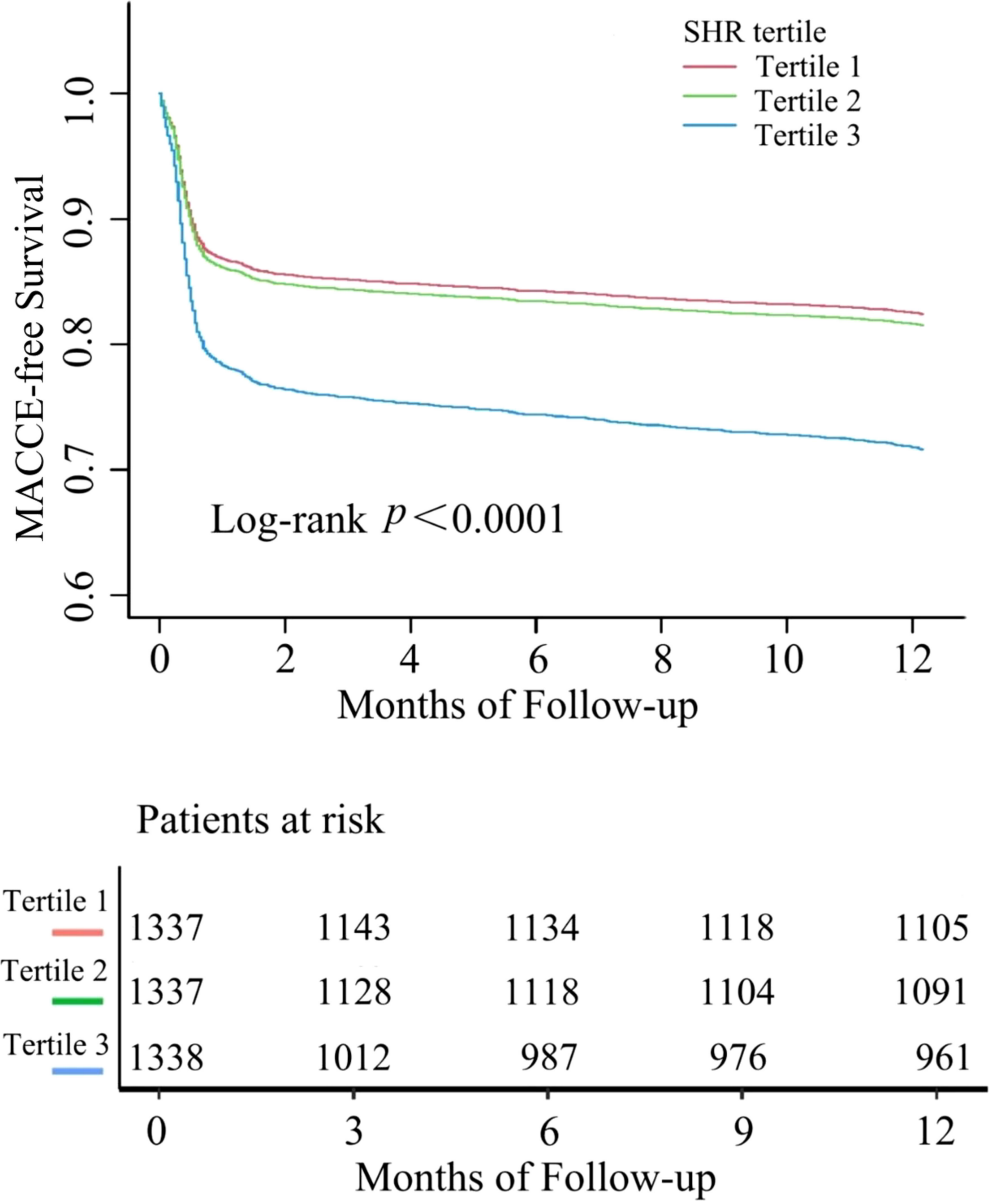
Kaplan–Meier survival curve for MACCE in patients with AMI according to SHR tertiles. MACCE, major adverse cardiovascular and cerebrovascular events.

During a 1-year follow-up, the crude incidence of MACCE was 21.46% (861/4012), including 5.71% all-cause mortality (229), 13.04% rehospitalizations for heart failure (523), 1.47% nonfatal strokes (59), 1.65% myocardial infarctions (66), and 4.46% rehospitalization for angina (179)(**Table 2**). To show the outcomes of patients in different SHR groups, we generated the Kaplan-Meier survival plots. As show in **Figure 2**, the MACCE-free survival is the lowest in tertile 3 group (log-rank *p <*0.001). **Table 3** shows the three Cox regression models used to evaluate the correlation between SHR and 1-year MACCE and all-cause mortality. For MACCE, whether SHR was considered as a categorical or continuous variable, remained significant after adjusting for confounders. Without adjusting for any covariates, for per 1 unit change increase the risk of incident MACCE by 154% (HR=2.54, 95%CI: 2.07-3.12), Compared with subjects in the lowest tertile, the HR for MACCE was 1.06 (95%CI: 0.88-1.26) and 1.72 (95%CI: 1.46, 2.03) in the middle and highest tertile, respectively. The increased risk of MACCE from tertile 1 to tertile 3 was statistically significant (*p* for trend<0.001). A similar pattern was observed in other three adjusted models. In model 1, age, sex, and BMI were adjusted, per 1 unit increase: HR=2.40, 95%CI: 1.96-2.94; Tertile 2: HR=1.06, 95%: CI 0.88-1.26; Tertile 3: HR=1.70, 95%CI: 1.45-2.00; *p* for trend<0.001. In model 2, age, sex, BMI, DM, hypertension, prior CVD, hyperlipemia, prior CAD, current smoking was adjusted, per 1 unit increase: HR=2.40, 95% CI: 1.95-2.94; Tertile 2: HR=1.09, 95%CI: 0.91-1.31; Tertile 3: HR=1.71, 95%CI: 1.45-2.01; *p* for trend<0.001. In the model 3, after adjusting for age, sex, BMI, DM, hypertension, prior CVD, hyperlipemia, prior CAD, current smoking, hs-CRP, cTNI, Scr, UA, D-D, ACEI/ARB, GS, PPCI, per 1 unit: HR=2.18, 95%CI: 1.64-2.89; Tertile 2: HR=1.06, 95%CI: 0.83-1.35; Tertile 3: HR=1.67, 95% CI:1.34-2.09; *p* for trend<0.001 respectively.

**Table 2:** Incidence of 1-Year clinical outcomes by SHR tertiles.

Outcome	Total (n=4012)	Tertile 1 (n=1337)	Tertile 2 (n=1337)	Tertile 3 (n=1338)	*P*-value
MACCE	861 (21.46%)	235 (17.58%)	248 (18.55%)	378 (28.25%)	<0.001
All-cause mortality	229 (5.71%)	58 (4.34%)	60 (4.49%)	111 (8.30%)	<0.001
Rehospitalization for heart failure	523 (13.04%)	130 (9.72%)	137 (10.25%)	256 (19.13%)	<0.001
Nonfatal stroke	59 (1.47%)	13 (0.97%)	14 (1.05%)	32 (2.39%)	0.003
Nonfatal myocardial infarction	66 (1.65%)	22 (1.65%)	22 (1.65%)	22 (1.64%)	0.988
Rehospitalization for angina	179 (4.46%)	56 (4.19%)	61 (4.56%)	62 (4.63%)	0.836

SHR, stress hyperglycemia ratio; MACCE, major adverse cardiovascular and cerebrovascular events.

**Table 3:** Multivariate Cox Regression analysis for one-year clinical outcomes.

	HR (95%CI) *P* value			
	Non-adjusted	Model 1	Model 2	Model 3
MACCE				
SHR per 1 unit	2.54 (2.07, 3.12) <0.0001	2.40 (1.96, 2.94) <0.0001	2.40 (1.95, 2.94) <0.0001	2.18 (1.64, 2.89) <0.0001
SHR per 1 SD increase	1.32 (1.24, 1.40) <0.0001	1.29 (1.22, 1.37) <0.0001	1.29 (1.22, 1.37) <0.0001	1.26 (1.16, 1.37) <0.0001
SHR tertile				
T1	Ref.	Ref.	Ref.	Ref.
T2	1.06 (0.88, 1.26) 0.5412	1.06 (0.88, 1.26) 0.5445	1.09 (0.91, 1.31) 0.3335	1.06 (0.83, 1.35) 0.6536
T3	1.72 (1.46, 2.03) <0.0001	1.70 (1.45, 2.00) <0.0001	1.71 (1.45, 2.01) <0.0001	1.67 (1.34, 2.09) <0.0001
*P* for trend	<0.0001	<0.0001	<0.0001	<0.0001
All-cause mortality				
SHR per 1 unit	4.15 (2.87, 6.00) <0.0001	3.66 (2.55, 5.26) <0.0001	3.65 (2.52, 5.29) <0.0001	3.11 (1.77, 5.46) <0.0001
SHR per 1 SD increase	1.52 (1.36, 1.70) <0.0001	1.47 (1.32, 1.63) <0.0001	1.46 (1.31, 1.63) <0.0001	1.40 (1.18, 1.65) <0.0001
SHR tertile				
T1	Ref.	Ref.	Ref.	Ref.
T2	1.04 (0.72, 1.49) 0.8460	1.03 (0.72, 1.48) 0.8750	1.07 (0.74, 1.53) 0.7266	0.90 (0.51, 1.57) 0.7020
T3	2.03 (1.48, 2.79) <0.0001	2.00 (1.45, 2.75) <0.0001	2.02 (1.47, 2.79) <0.0001	1.67 (1.02, 2.73) 0.0415
*P* for trend	<0.0001	<0.0001	<0.0001	0.020

SHR, stress hyperglycemia index; BMI, body mass index; ACEI/ARB, angiotensin-converting enzyme inhibitor/angiotensin receptor blocker; GS: Gensini score; D-D: D-dimer.

Non-adjusted model: No covariates were adjusted.

Model 1 adjust for: age, sex, BMI.

Model 2 adjust for: age, sex, BMI, DM, hypertension, previous CVD, hyperlipemia, previous CAD, current smoking.

Model 3 adjust for: age, sex, BMI, DM, hypertension, previous CVD, hyperlipemia, previous CAD, current smoking, hs-CRP, cTNI, Scr, PPCI, ACEI/ARB, GS, UA, D-D.

Moreover, we explored the relationships between the SHR and 1-year all-cause mortality, indicating a similar pattern to the connection between the SHR and all-cause mortality (**Table 3**). Despite adjusting for confounders, the significance of SHR remained whether it was categorized or treated as a continuous variable. Without adjusting for any covariates, a per 1 unit SHR change was associated with a 315% increased risk of incident all-cause mortality (HR = 4.15, 95%CI:2.87-6.00). When compared to individuals in the lowest tertile, those in the middle tertile had a HR of 1.04 (95%CI: 0.72, 1.49) and those in the highest tertile had a HR of 2.03 (95%CI: 1.48-2.79) for all-cause mortality. The increased risk of all-cause mortality from tertile 1 to tertile 3 was statistically significant (*p* for trend<0.001). A similar pattern was observed in other three adjusted models. In model 1, age, sex, and BMI were adjusted, per 1 unit increase: HR=3.66, 95%CI: 2.55-5.26; Tertile 2: HR=1.03, 95% CI: 0.72-1.48; Tertile 3: HR=1.70, 95% CI 1.45-2.75; *p* for trend<0.001. In model 2, age, sex, BMI, DM, hypertension, prior CVD, hyperlipemia, prior CAD, current smoking was adjusted, per 1 unit: HR=3.65, 95%CI: 2.52-5.29; Tertile 2: HR=1.07, 95%CI: 0.74-1.53; Tertile 3: HR=2.02, 95%CI: 1.47-2.79; *p* for trend<0.001. In the model 3, after adjusting for age, sex, BMI, DM, hypertension, prior CVD, hyperlipemia, prior CAD, current smoking, hs-CRP, cTNI, Scr, UA, D-D, ACEI/ARB, GS, PPCI, per 1 unit increase: HR=3.11, 95%CI: 1.77-5.46; Tertile 2: HR= 0.90, 95% CI: 0.51-1.57; Tertile 3: HR=1.67, 95%CI: 1.02-2.73; p for trend=0.020, respectively.

In the further, we conducted additional Cox regression analyses to examine the associations between individual components of MACCE and SHR. For Heart Failure Rehospitalization for heart failure (RHF): SHR showed strong positive relationships with 1-year RHF risk. In all multivariate Cox regression models, positive correlations were observed between them: model 1 (model 1: HR=3.01, 95% CI:2.35-3.87), model 2 (model 2: HR=2.95, 95% CI:2.29-3.80), and model 3 (HR=2.31, 95% CI: 1.76-3.02). Interestingly, the incidence of RHF increased by 131% for every unit rise in SHR after correcting for possible confounders (model 3). Further dividing SHR into tertile and using the T1 group as the reference, we examine the connection between SHR and RHF. In Model 3, participants in the T3 group of SHR had a 88% higher risk of all-cause mortality compared to those in the T1 group (HR: 1.88, 95% CI: 1.48-2.39) after controlling for age, sex, BMI, DM, hypertension, prior CVD, hyperlipemia, prior CAD, current smoking, hs-CRP, cTNI, Scr, UA, D-D, ACEI/ARB, GS, PPCI. A statistically significant trend of increasing RHF risk was observed from T1 to T3 group (P for trend<0.001) (**Supplementary Material**: **Supplementary Table S1**). For nonfatal stroke, the SHR demonstrated a significant positive association with 1-year nonfatal stroke risk. When SHR was considered a continuous variable, the incidence of nonfatal stroke increase by 175% for per 1 unit rise in SHR after adjusting for potential confounders (model 3: HR=2.75, 95% CI: 1.21-6.22). When SHR was treated as a categorical variable, individuals in the T3 group exhibited a 166% higher risk of nonfatal stroke compared to those in the T1 group in Model 3 (HR=2.66, 95% CI: 1.25-5.64, P=0.0108), with a statistically significant linear trend (*P* for trend=0.0089) (**Supplementary Material**: **Supplementary Table S2**). However, Nonfatal MI and rehospitalization for angina did not reach statistical significance in multivariable models (**Supplementary Tables S3**-**S4**), likely due to lower event rates (Nonfatal MI: 1.65%, rehospitalization for angina: 4.46%) reducing statistical power.

### 3.3 Non-linear relationships between the SHR and 1-year clinical outcomes

**Figure 3:**
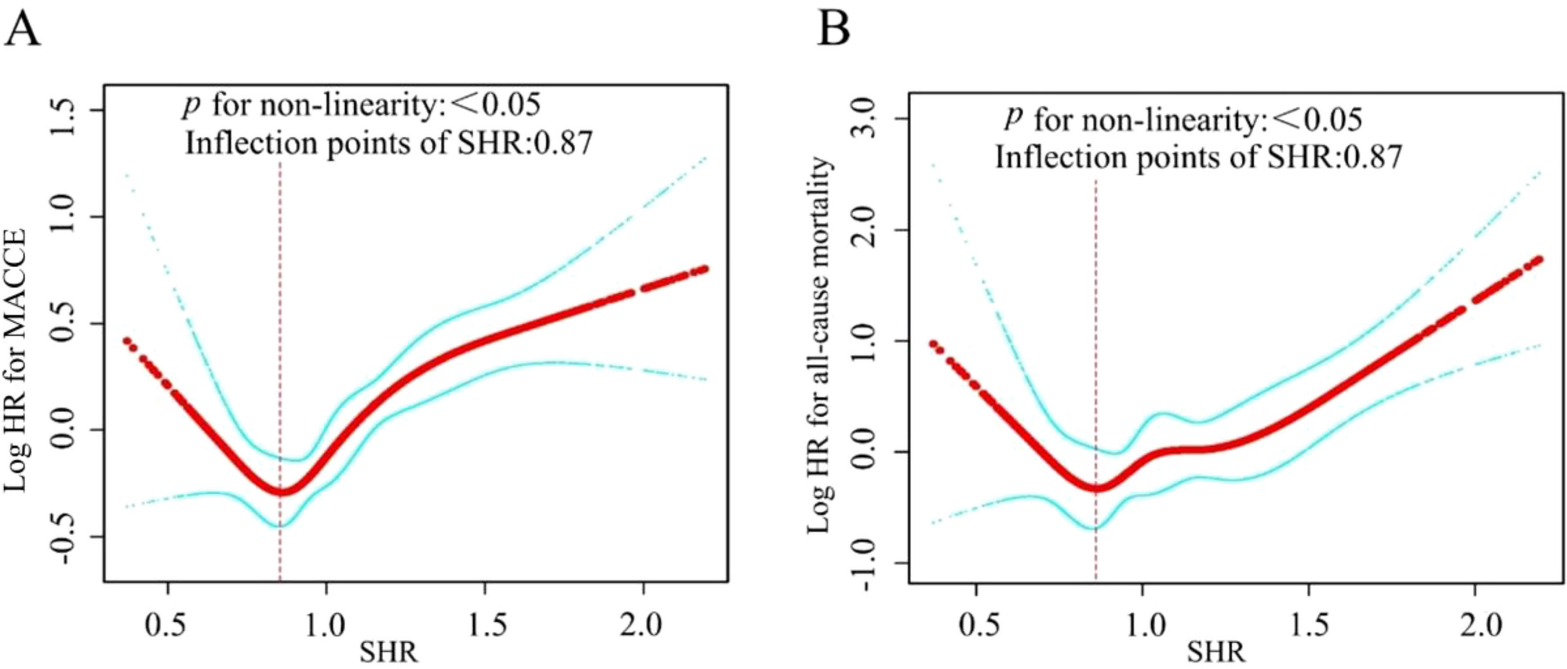
In this study, we used a smoothed curve - fitting method to analyze the relationship between SHR and MACCE **(A)** and all-cause mortality **(B)** in AMI patients. The red curve shows the risk, and the blue one its 95% confidence interval. Curve plot demonstrated the non-linear relationship between SHR and MACCE **(A)** and all-cause mortality **(B)** among all participants, adjusted for various factors including age, sex, BMI, DM, hypertension, previous CVD, hyperlipemia, previous CAD, current smoking, hs-CRP, cTNI, Scr, PPCI, ACEI/ARB, GS, UA, D-D.

The nonlinearity of the association between SHR and 1-year clinical outcomes was discerned through the application of a Cox proportional hazards regression model with restricted cubic spline functions, as depicted in **Figure 3**. Subsequently, the most suitable model was ascertained via the loglikelihood ratio test, the results of which are detailed in **Table 4**, yielding a *p* -value of less than 0.05. Employing RCS analysis with four knots, we identified the J-shaped relationship between SHR and outcomes and determined the inflection point (SHR=0.87). Notably, when the SHR was > 0.87, we observed that a 1-unit increase in the SHR was associated with a sharply increased in the risk of MACCE (HR 2.64, 95% CI 1.91–3.65). When the SHR was < 0.87, the risk of MACCE was not significantly associated with changes in the SHR. For all-cause mortality, when the SHR index was > 0.87, it showed a significant positive association with the risk of all-cause mortality (HR 4.26, 95% CI 2.30–7.86).

**Table 4:** Threshold effect analysis of SHR and one-year clinical outcomes using Piece-wise linear regression.

	MACCE		All-cause mortality	
Model I	HR (95%CI)	P value	HR (95%CI)	P value
One line effect	2.18 (1.64, 2.89)	<0.0001	3.11 (1.77, 5.46)	<0.0001
Model II				
Turn point (K)	0.87		0.87	
SHR < K	0.39 (0.09, 1.67)	0.2032	0.08 (0.00, 1.80)	0.1130
SHR>K	2.64 (1.91, 3.65)	<0.0001	4.26 (2.30, 7.86)	<0.0001
*P* value for LRT test*	0.024*		0.035*	

SHR, stress hyperglycemia index; BMI, body mass index; ACEI/ARB, angiotensin-converting enzyme inhibitor/angiotensin receptor blocker; GS: Gensini score; D-D: D-dimer.

Data were presented as OR (95% CI) P value; Model I, linear analysis; Model II, non-linear analysis. CI confidence interval, OR odds ratio, LRT logarithm likelihood ratio test. *P < 0.05 indicates that model II is significantly different from Model I. Adjusted for age, sex, BMI, DM, hypertension, previous CVD, hyperlipemia, previous CAD, current smoking, hs-CRP, cTNI, Scr, PPCI, ACEI/ARB, GS, UA, D-D.

### 3.4 Subgroup analysis

Subgroup analyses were performed to evaluate the associations of SHR with MACCE and mortality in different populations according to age (≤60 years, 60-70 years, ≥ 70 years), sex (female, male), BMI (≤26, >26), and diabetes status (yes, no), hypertension (yes, no), current smoking (yes, no), LVEF (≤40, >40), number of diseased vessels (SVD, MVD). The relationship between SHR and MACCE and all-cause mortality among AMI patients was consistent across the various subgroups, as depicted in **Table 5**. There was no significant interaction effect between SHR and stratified variables.

**Table 5:** Stratified analyses of the associations between SHR and one-year outcomes.

Characteristic	MACCE				All -cause death			
	N (%)	HR (95%CI)	*P* -value	*p-int*	N (%)	HR (95%CI)	*P* -value	*p-int*
Sex				0.1328				0.1887
Male	3025	1.89 (1.35, 2.66)	0.0002		3025	2.74 (1.31, 5.74)	0.0075	
Female	987	3.00 (1.82, 4.92)	<0.0001		987	5.81 (2.48, 13.63)	<0.0001	
BMI				0.2638				0.1346
≤26	2686	2.41 (1.72,3.38)	<0.0001		2686	4.38 (2.37, 8.12)	0.0001	
>26	1317	1.71 (1.03, 2.85)	0.0391		1317	1.61 (0.48, 5.37)	0.4396	
Age				0.6328				0.6822
<=60	1796	1.83 (1.08, 3.10)	0.0243		1796	5.58 (1.17, 26.62)	0.0309	
60-70	1255	2.13 (1.36, 3.32)	0.0009		1255	2.63 (1.03, 6.70)	0.0434	
>=70	961	2.58 (1.59, 4.16)	0.0001		961	3.81 (1.80, 8.07)	0.0005	
LVEF				0.0898				0.1879
<=40	389	3.24 (1.82, 5.76)	<0.0001		389	5.58 (2.01, 15.54)	0.001	
>40	3453	1.82 (1.31, 2.55)	0.0004		3453	2.45 (1.22, 4.93)	0.0116	
Hypertension				0.7525				0.3022
No	1157	2.30 (1.48, 3.59)	0.0002		1157	4.99 (2.16, 11.51)	0.0002	
Yes	1289	2.10 (1.47, 3.01)	<0.0001		1289	2.83 (1.39, 5.80)	0.0043	
DM				0.5196				0.8376
No	1816	2.36 (1.63, 3.41)	<0.0001		1816	3.40 (1.67, 6.91)	0.0007	
Yes	630	1.96 (1.27, 3.02)	0.0024		630	3.83 (1.56, 9.41)	0.0034	
NDV				0.3993				0.3366
SVD	487	1.64 (0.79, 3.37)	0.1813		487	1.91 (0.49, 7.36)	0.3497	
MVD	1959	2.28 (1.68, 3.10)	<0.0001		1959	3.89 (2.11, 7.18)	<0.0001	
Current smoking				0.1554				0.112
No	938	2.74 (1.80, 4.18)	<0.0001		938	5.36 (2.56, 11.21)	<0.0001	
Yes	1508	1.84 (1.27, 2.67)	0.0014		1508	2.26 (1.00, 5.10)	0.049	

NDV, number of diseased vessels; SVD, single vessel disease; MVD, multivessel disease; BMI, Body mass index.

Subgroup analyses were conducted to test the stability of the relationship between SHR and one-year outcomes in AMI patients.

Adjusted for age, sex, BMI, DM, hypertension, previous CVD, hyperlipemia, previous CAD, current smoking, hs-CRP, cTNI, Scr, PPCI, ACEI/ARB, GS, UA, D-D.

## 4 Discussion

This retrospective cohort study, encompassing a large sample of 4,012 AMI patients monitored over a one-year period, identified a significant association between SHR and the incidence of one-year MACCE and all-cause mortality, even after adjusting for potential confounders. A J-shaped relationship was observed between SHR values and these outcomes, with threshold effect analysis pinpointing an inflection point at 0.87, a significantly higher risk for both scenarios was reported after this turning point subsequently. Subgroup analyses remained consistent across age, sex, BMI, LVEF, Hypertension, DM, NDV, current smoking. In conclusion, our research demonstrates that SHR serves as a robust prognostic indicator for predicting one-year MACCE and all-cause mortality in AMI patients, potentially guiding the refinement of preventive strategies for this population.

Stress-induced hyperglycemia (SIH) represents a prevalent clinical condition characterized by a complex pathophysiological framework involving multiple mechanisms, including neural activation, hormonal dysregulation, and insulin resistance phenomena (21–23). While moderate hyperglycemia may serve as an adaptive response to acute stress (24) excessive elevations impair physiological homeostasis. The development of SIH arises from a multifaceted interplay of acute metabolic alterations, such as enhanced gluconeogenesis, excessive adrenergic stimulation, insulin resistance, and overactivation of counter-regulatory hormones (e.g., catecholamines, cortisol, and proinflammatory cytokines) (25). Paradoxically, sustained SIH perpetuates a vicious cycle through amplified inflammatory cytokine release, oxidative stress, endothelial dysfunction, prothrombotic states, and ischemia-reperfusion injury – all exacerbating myocardial damage (26). Clinically, elevated admission SHR reflects profound inflammatory and hemodynamic disturbances in AMI patients, particularly those with complications like cardiogenic shock or systemic infection. Acute glycemic variability further correlates with plaque destabilization, myocardial infarct expansion, and impaired ventricular function (27), collectively predicting adverse outcomes. Contemporary studies have demonstrated significant associations between SHR and intracoronary thrombus burden, as well as no-reflow phenomenon (22, 28), potentially explaining the elevated mortality and cardiogenic shock rates observed in severe stress hyperglycemia. These findings underscore the critical importance of rigorous SIH assessment for early risk stratification and informed clinical decision-making.

The use of admission blood glucose (ABG) levels to assess stress hyperglycemia remains widespread; however, this single time-point measurement may fail to adequately capture acute glycemic fluctuations. To address this limitation, the acute-to-chronic glycemic ratio offers a more comprehensive approach for evaluating stress-induced glycemic variability compared to relying solely on admission glucose. Building on this concept, Roberts et al. proposed the SHR, a novel metric calculated as ABG adjusted for chronic glycemic status via HbA1c to better quantify stress hyperglycemia (10). This index quantifies acute glycemia using ABG while approximating chronic glycemia through HbA1c.Notably, SHR has been independently linked to cerebral edema following acute cerebral infarction (29), in-hospital pulmonary infection risk (30), and the severity of coronary artery disease and thrombus burden (31). Multiple studies involving acute coronary syndrome (ACS) patients have further validated SHR’s prognostic value for adverse outcomes. For instance, Xu et al. demonstrated that SHR significantly improves the predictive capacity of the TIMI risk score for 30-day mortality in STEMI patients (12). Similarly, Sia et al. identified SHR as an independent predictor of one-year mortality in ST-elevation myocardial infarction patients, regardless of diabetes status (32). Collectively, these findings underscore SHR’s critical role in risk stratification for ACS populations. A key strength of our study, distinguishing it from prior research, lies in its inclusion of 4,012 AMI patients and systematic implementation of one-year follow-up for MACCE.

The present study provided evidence of the independent association between SHR and 1-year adverse cardiovascular events, including MACCE, all-cause mortality in AMI patients. Furthermore, restricted cubic spline (RCS) analysis delineated a J-shaped relationship between SHR and both one-year MACCE and all-cause mortality, with HRs for MACCE and mortality increasing significantly when SHR exceeded > 0.87. These observations align with prior research to a certain extent. Yang et al. identified a J-shaped or U-shaped association between SHR and poor prognosis in ACS patients, mirroring our findings (33). Roberts et al. described a predominantly J-shaped relationship between SHR and critical illness, consistent with our research (10). A large-scale study by Sia et al. demonstrated an independent association between the SHR and one-year mortality in patients with STEMI, irrespective of their diabetic status. The study also highlighted the superiority of SHR over admission glucose in predicting one-year mortality (32). Additionally, stress hyperglycemia, as measured by glucose/glycated albumin ratio, exhibited a significant U-shaped relationship only in ACS patients with diabetes mellitus, as opposed to those without diabetes (34), which partially aligns with our findings. Our study uniquely investigates the J-shaped association between SHR and 1-year prognosis, identifying a critical threshold (SHR=0.87) that may guide targeted therapeutic interventions (15). Elevated stress hyperglycemia ratio (SHR) has been consistently associated with adverse clinical outcomes, primarily through acute hyperglycemia-induced oxidative stress and vascular endothelial dysfunction. This pathophysiological cascade increases cardiovascular vulnerability and predicts poorer prognosis. Conversely, lower SHR levels in diabetic patients may reflect better glycemic control stability and preserved metabolic adaptability, potentially serving as a protective factor against acute glycemic fluctuations. Despite the risks associated with chronic hyperglycemia, the diminished effect of acute glycemic fluctuations in these patients implies the maintenance of stress response capacity and intact insulin signaling pathways, which are essential determinants of cardiovascular resilience. This study included 4012 patients with AMI, covering different subgroups such as BMI, diabetes status, and number of diseased vessels, which verified the universality of the association between SHR and outcomes.

The precise mechanisms linking SHR to adverse outcomes in ACS patients remain incompletely understood. Nevertheless, several plausible mechanisms have been proposed (1): Stress-induced hyperglycemia (SIH) exacerbates insulin resistance (IR) while triggering excessive release of counter-regulatory hormones, including catecholamines, cortisol, glucagon, and growth hormone. Catecholamines directly suppress insulin secretion, impair glucose transporter function (e.g., GLUT4), and enhance hepatic gluconeogenesis, collectively worsening peripheral IR (35); (2) Elevated glucose levels promote reactive oxygen species (ROS) overproduction, initiating inflammatory cascades and oxidative damage. This dual insult accelerates both microvascular (e.g., retinopathy, nephropathy) and macrovascular (e.g., atherosclerosis) complications in diabetic patients (36); (3) Chronic hyperglycemia compromises endothelial nitric oxide synthase (eNOS) activity, reducing vasodilatory capacity. This endothelial impairment is further aggravated by SHR elevation, creating a pro-atherogenic milieu (26); (4) SHR-mediated endothelial injury exposes subendothelial collagen, activating platelets and coagulation pathways. Subsequent platelet adhesion, aggregation, and fibrin deposition heighten thrombotic risk (37); (5) High SHR levels correlate with elevated plasminogen activator inhibitor-1 (PAI-1), a key inhibitor of clot dissolution. Notably, improved glycemic control reduces PAI-1 concentrations, demonstrating the reversibility of SHR-associated fibrinolytic dysfunction (38).

Despite the well-established link between stress-induced hyperglycemia and the prognosis of AMI, the most effective treatment strategies for stress hyperglycemia are yet to be determined. Clinical trials assessing glucose-lowering therapies targeting specific glucose levels has yielded mixed outcomes. For example, the DIGAMI study demonstrated that intensive insulin therapy lowered overall mortality in AMI patients with stress hyperglycemia, independent of their diabetic status (39). Conversely, a meta-analysis encompassing three studies reported only marginal benefits from intensive glucose management in diabetic individuals suffering from AMI, while also highlighting a significant rise in the occurrence of severe hypoglycemia (40). Given the pressing necessity for innovative, there is a pressing need for novel targeted therapeutic strategies to combat stress-induced hyperglycemia, particularly considering the increasingly recognized protective effects of modern oral antidiabetic medications like sodium-glucose cotransporter-2 inhibitors (SGLT2-Is) and glucagon-like peptide-1 receptor agonists (GLP-1 RAs) (41). In patients with AMI who have received long-term treatment with SGLT2 inhibitors, a notable reduction in inflammatory responses, infarct sizes, and instances of stress-induced hyperglycemia has been observed (42). The cardioprotective benefits of SGLT2-Is have been demonstrated to go beyond merely controlling glycemia. Similar beneficial outcomes have been observed with GLP-1 RAs (43). Considering the SHR has been identified as a more robust predictor of adverse prognosis, it is proposed that future research should consider the application of stratified glycemic targets based on SHR levels, rather than relying solely on absolute glucose measurements, for the management of SIH.

The strengths of this study include a large sample size and a comprehensive assessment of the dose-response relationship between SHR and 1-year MACCE/all-cause mortality in AMI patients using restricted cubic spline (RCS) analysis. Importantly, we successfully identified inflection points within the J-shaped relationship, providing actionable thresholds for clinical risk stratification. However, several limitations warrant consideration. First, the study population was drawn from a single center and exclusively comprised Asian patients, raising concerns about generalizability. While rigorous adjustments were made, the potential for selection bias remains, necessitating validation through prospective multicenter studies. Second, despite extensive covariate adjustment, residual confounding from unmeasured factors (e.g., genetic predispositions, undiagnosed comorbidities) or SHR-influencing variables (e.g., nutritional status, inflammatory markers) cannot be excluded. Third, the absence of data on diabetes duration, glycemic control strategies, and post-discharge medication adherence precluded analysis of interactions between SHR and glucose-lowering therapies. Finally, in our only focus on 1-year outcomes highlights the need for extended longitudinal studies to evaluate the durability of SHR’s prognostic utility. Our study period (2016–2019) occurred before the common use of SGLT2 inhibitors and GLP-1 RAs for reducing cardiovascular risk in AMI patients. Subsequent research should assess whether SHR maintains its prognostic significance in the era of contemporary glucose-lowering treatments. Despite those constraints, our findings necessitate cautious interpretation. Future research, encompassing a more diverse population, extended follow-up durations, and prospective evaluations, is imperative to validate SHR’s significance in AMI patient outcomes.

In summary, the data presented strongly indicate a notable association between SHR and the clinical outcomes of AMI patients, emphasizing its prognostic significance for MACCE and all-cause mortality. Consequently, it is proposed that SHR may function as an efficacious and straightforward indicator for risk stratification and early intervention in the AMI patients.

## Data Availability

The raw data supporting the conclusions of this article will be made available by the authors, without undue reservation.
