# Supplementary material for: The association between stress hyperglycemia ratio and 1-year outcomes in patients with acute myocardial infarction: a retrospective large sample cohort study
Source: Front Endocrinol (Lausanne). 2025 Apr 15;16:1586541. doi: 10.3389/fendo.2025.1586541 (PMC12037398; doi:10.3389/fendo.2025.1586541)
Supplement: Supplementary file 1 [file DataSheet1.pdf]

**Supplementary Information to:**

# The association between Stress Hyperglycemia ratio and 1-year outcomes in patients with acute myocardial infarction: A retrospective large sample cohort study

Yan Ning[1,*,†], Peng Wu[1,2,†], Zhengjun Zhang[1], Mohan Wang[1]， MaJuan[1], Ali Ma[2], Dapeng Chen[1], Xueping Ma [1], Xiaocheng Li [3,*]

**Affiliations:**

[1] Heart Centre & Department of Cardiovascular Diseases, General Hospital of Ningxia Medical University, Yinchuan, 750004, People's Republic of China

[2] First Clinical College, Ningxia Medical University, Yinchuan, China

[3] Institute of Basic Medical Sciences, Xi'an Medical University, Xi'an, China

*Corresponding author:

Xiaocheng Li, Institute of Basic Medical Sciences, Xi'an Medical University, Xi'an 710021, Shaanxi, China, cyclone9218@163.com.

Ning Yan, Heart Centre & Department of Cardiovascular Diseases, General Hospital of Ningxia Medical University, Yinchuan 750004, Ningxia, China. Tel: +8615719591301, yanning169@yahoo.com

**Table S1** Multivariable Cox Regression Analysis for One-Year Rehospitalization for Heart Failure

| | HR (95%CI) *P* value | | | |
|---|---|---|---|---|
| | Non-adjusted | Model 1 | Model 2 | Model 3 |
| **Rehospitalization for HF** | | | | |
| SHR per 1 unit | 3.33 (2.59, 4.29) <0.0001 | 3.01 (2.35, 3.87) <0.0001 | 2.95 (2.29, 3.80) <0.0001 | 2.31 (1.76, 3.02) <0.0001 |
| SHR per 1 SD increase | 1.43 (1.32, 1.54) <0.0001 | 1.38 (1.29, 1.49) <0.0001 | 1.38 (1.28, 1.48) <0.0001 | 1.28 (1.18, 1.39) <0.0001 |
| SHR tertile | | | | |
| T1 | Ref. | Ref. | Ref. | Ref. |
| T2 | 1.05 (0.83, 1.34) 0.6720 | 1.05 (0.83, 1.34) 0.6769 | 1.10 (0.87, 1.40) 0.4261 | 1.16 (0.89, 1.51) 0.2869 |
| T3 | 2.08 (1.69, 2.57) <0.0001 | 2.03 (1.64, 2.50) <0.0001 | 2.03 (1.64, 2.51) <0.0001 | 1.88 (1.48, 2.39) <0.0001 |
| *P* for trend | <0.0001 | <0.0001 | <0.0001 | <0.0001 |

**Abbreviations**: SHR, stress hyperglycemia index; BMI, body mass index; ACEI/ARB, angiotensin-converting enzyme inhibitor/angiotensin receptor blocker; GS: Gensini score; D-D: D-dimer.

Non-adjusted model: No covariates were adjusted.

Model 1 adjust for: age, sex, BMI.

Model 2 adjust for: age, sex, BMI, DM, hypertension, previous CVD, hyperlipemia, previous CAD, current smoking.

Model 3 adjust for: age, sex, BMI, DM, hypertension, previous CVD, hyperlipemia, previous CAD, current smoking, hs-CRP, cTNI, Scr, PPCI, ACEI/ARB, GS, UA, D-D.

**Table S2** Multivariable Cox Regression Analysis for One-Year Nonfatal stroke

| | HR (95%CI) *P* value | | | |
|---|---|---|---|---|
| | Non-adjusted | Model 1 | Model 2 | Model 3 |
| **Nonfatal stroke** | | | | |
| SHR per 1 unit | 4.21 (2.03, 8.74)<0.0001 | 4.33 (2.10, 8.96) <0.0001 | 4.78 (2.26, 10.12)<0.0001 | 2.75 (1.21, 6.22) 0.0152 |
| SHR per 1 SD increase | 1.53 (1.23, 1.89)<0.0001 | 1.54 (1.24, 1.91) <0.0001 | 1.59 (1.27, 1.98)<0.0001 | 1.35 (1.06, 1.71) 0.0152 |
| SHR tertile | | | | |
| T1 | Ref. | Ref. | Ref. | Ref. |
| T2 | 1.08 (0.51, 2.29) 0.8475 | 1.09 (0.51, 2.32) 0.8227 | 1.14 (0.54, 2.44) 0.7280 | 1.44 (0.61, 3.40) 0.4071 |
| T3 | 2.63 (1.38, 5.00) 0.0033 | 2.72 (1.43, 5.20) 0.0024 | 2.80 (1.46, 5.37) 0.0020 | 2.66 (1.25, 5.64) 0.0108 |
| *P* for trend | 0.0015 | 0.0011 | 0.0010 | 0.0089 |

**Abbreviations**: SHR, stress hyperglycemia index; BMI, body mass index; ACEI/ARB, angiotensin-converting enzyme inhibitor/angiotensin receptor blocker; GS: Gensini score; D-D: D-dimer.

Non-adjusted model: No covariates were adjusted.

Model 1 adjust for: age, sex, BMI.

Model 2 adjust for: age, sex, BMI, DM, hypertension, previous CVD, hyperlipemia, previous CAD, current smoking.

Model 3 adjust for: age, sex, BMI, DM, hypertension, previous CVD, hyperlipemia, previous CAD, current smoking, hs-CRP, cTNI, Scr, PPCI, ACEI/ARB, GS, UA, D-D.

**Table S3** Multivariable Cox Regression Analysis for One-Year Nonfatal MI

| | HR (95%CI) *P* value | | | |
|---|---|---|---|---|
| | Non-adjusted | Model 1 | Model 2 | Model 3 |
| **Nonfatal MI** | | | | |
| SHR per 1 unit | 1.17 (0.51, 2.71) 0.7137 | 1.15 (0.50, 2.66) 0.7391 | 1.22 (0.52, 2.85) 0.6416 | 0.79 (0.26, 2.45) 0.6856 |
| SHR per 1 SD increase | 1.05 (0.82, 1.34) 0.7137 | 1.04 (0.81, 1.33) 0.7391 | 1.06 (0.83, 1.36) 0.6416 | 0.93 (0.67, 1.30) 0.6856 |
| SHR tertile | | | | |
| T1 | Ref. | Ref. | Ref. | Ref. |
| T2 | 1.01 (0.56, 1.82) 0.9834 | 1.01 (0.56, 1.82) 0.9741 | 1.03 (0.57, 1.86) 0.9269 | 1.25 (0.58, 2.70) 0.5767 |
| T3 | 1.11 (0.61, 2.00) 0.7350 | 1.10 (0.61, 2.00) 0.7418 | 1.13 (0.62, 2.05) 0.6920 | 1.26 (0.57, 2.78) 0.5697 |
| *P* for trend | 0.7369 | 0.7436 | 0.6946 | 0.5691 |

**Abbreviations**: SHR, stress hyperglycemia index; BMI, body mass index; ACEI/ARB, angiotensin-converting enzyme inhibitor/angiotensin receptor blocker; GS: Gensini score; D-D: D-dimer.

Non-adjusted model: No covariates were adjusted.

Model 1 adjust for: age, sex, BMI.

Model 2 adjust for: age, sex, BMI, DM, hypertension, previous CVD, hyperlipemia, previous CAD, current smoking.

Model 3 adjust for: age, sex, BMI, DM, hypertension, previous CVD, hyperlipemia, previous CAD, current smoking, hs-CRP, cTNI, Scr, PPCI, ACEI/ARB, GS, UA, D-D.

**Table S4** Multivariable Cox Regression Analysis for One-Year Rehospitalization for angina

| | HR (95%CI) *P* value | | | |
|---|---|---|---|---|
| | Non-adjusted | Model 1 | Model 2 | Model 3 |
| **Rehospitalization for angina** | | | | |
| SHR per 1 unit | 1.11 (0.67, 1.85) 0.6803 | 1.11 (0.66, 1.85) 0.7026 | 1.09 (0.66, 1.82) 0.7369 | 1.29 (0.72, 2.31) 0.3982 |
| SHR per 1 SD increase | 1.03 (0.89, 1.20) 0.6803 | 1.03 (0.89, 1.20) 0.7026 | 1.03 (0.88, 1.19) 0.7369 | 1.08 (0.91, 1.28) 0.3982 |
| SHR tertile | | | | |
| T1 | Ref. | Ref. | Ref. | Ref. |
| T2 | 1.10 (0.76, 1.57) 0.6234 | 1.08 (0.75, 1.55) 0.6914 | 1.12 (0.78, 1.62) 0.5354 | 1.16 (0.75, 1.80) 0.5070 |
| T3 | 1.21 (0.84, 1.73) 0.3041 | 1.20 (0.83, 1.72) 0.3270 | 1.19 (0.83, 1.72) 0.3413 | 1.33 (0.86, 2.04) 0.2016 |
| *P* for trend | 0.3039 | 0.3267 | 0.3405 | 0.2008 |

**Abbreviations**: SHR, stress hyperglycemia index; BMI, body mass index; ACEI/ARB, angiotensin-converting enzyme inhibitor/angiotensin receptor blocker; GS: Gensini score; D-D: D-dimer.

Non-adjusted model: No covariates were adjusted.

Model 1 adjust for: age, sex, BMI.

Model 2 adjust for: age, sex, BMI, DM, hypertension, previous CVD, hyperlipemia, previous CAD, current smoking.

Model 3 adjust for: age, sex, BMI, DM, hypertension, previous CVD, hyperlipemia, previous CAD, current smoking, hs-CRP, cTNI, Scr, PPCI, ACEI/ARB, GS, UA, D-D.
